# Critical Role for the Advanced Glycation End‐Products Receptor in Pulmonary Arterial Hypertension Etiology

**DOI:** 10.1161/JAHA.112.005157

**Published:** 2013-02-22

**Authors:** Jolyane Meloche, Antony Courchesne, Marjorie Barrier, Sophie Carter, Malik Bisserier, Roxane Paulin, Jean‐François Lauzon‐Joset, Sandra Breuils‐Bonnet, Ève Tremblay, Sabrina Biardel, Christine Racine, Christian Courture, Pierre Bonnet, Susan M. Majka, Yves Deshaies, Frédéric Picard, Steeve Provencher, Sébastien Bonnet

**Affiliations:** 1 Pulmonary Hypertension Group of the Institut universitaire de cardiologie et de pneumologie de Québec, Laval University, Quebec City, Canada (J.M., A.C., M.B., M.B., R.P., S.B.B., T., S.P., B.); 2 Institut universitaire de cardiologie et de pneumologie de Québec, Quebec City, Canada (J.M., A.C., M.B., S.C., M.B., R.P., J.F.L.J., S.B.B., T., S.B., C.R., C.C., Y.D., F.P., S.P., B.); 3 Faculty of Medicine, University François Rabelais, Tours Cedex, France (P.B.); 4 Department of Medicine, Division of Allergy, Pulmonary and Critical Care Medicine, Vanderbilt University, Nashville, TN (S.M.M.)

**Keywords:** PPARγ, pulmonary hypertension, RAGE, remodeling, Sugen

## Abstract

**Background:**

Pulmonary arterial hypertension (PAH) is a vasculopathy characterized by enhanced pulmonary artery smooth muscle cell (PASMC) proliferation and suppressed apoptosis. This results in both increase in pulmonary arterial pressure and pulmonary vascular resistance. Recent studies have shown the implication of the signal transducer and activator of transcription 3 (STAT3)/bone morphogenetic protein receptor 2 (BMPR2)/peroxisome proliferator‐activated receptor gamma (PPARγ) in PAH. STAT3 activation induces BMPR2 downregulation, decreasing PPARγ, which both contribute to the proproliferative and antiapoptotic phenotype seen in PAH. In chondrocytes, activation of this axis has been attributed to the advanced glycation end‐products receptor (RAGE). As RAGE is one of the most upregulated proteins in PAH patients' lungs and a strong STAT3 activator, we hypothesized that by activating STAT3, RAGE induces BMPR2 and PPARγ downregulation, promoting PAH‐PASMC proliferation and resistance to apoptosis.

**Methods and Results:**

In vitro, using PASMCs isolated from PAH and healthy patients, we demonstrated that RAGE is overexpressed in PAH‐PASMC (6‐fold increase), thus inducing STAT3 activation (from 10% to 40% positive cells) and decrease in BMPR2 and PPARγ levels (>50% decrease). Pharmacological activation of RAGE in control cells by S100A4 recapitulates the PAH phenotype (increasing RAGE by 6‐fold, thus activating STAT3 and decreasing BMPR2 and PPARγ). In both conditions, this phenotype is totally reversed on RAGE inhibition. In vivo, RAGE inhibition in monocrotaline‐ and Sugen‐induced PAH demonstrates therapeutic effects characterized by PA pressure and right ventricular hypertrophy decrease (control rats have an mPAP around 15 mm Hg, PAH rats have an mPAP >40 mm Hg, and with RAGE inhibition, mPAP decreases to 20 and 28 mm Hg, respectively, in MCT and Sugen models). This was associated with significant improvement in lung perfusion and vascular remodeling due to decrease in proliferation (>50% decrease) and BMPR2/PPARγ axis restoration (increased by ≥60%).

**Conclusion:**

We have demonstrated the implications of RAGE in PAH etiology. Thus, RAGE constitutes a new attractive therapeutic target for PAH.

## Introduction

Pulmonary arterial hypertension (PAH) is a devastating disease of the pulmonary vasculature characterized by enhanced inflammation, vasoconstriction, and remodeling of small pulmonary arteries (PAs).^1^ This results in an increase in pulmonary vascular resistance and pressure, which first leads to right ventricle hypertrophy (RVH) and ultimately to right ventricular (RV) failure and death.^2–3^ PAH is still a lethal disease, and in fact, mortality rate is still 40% in 5 years.^4^ This is explained by lack of understanding of its physiopathology and by the absence of any medication targeting the vascular remodeling processes.

Remodeling of PAs in PAH is a result of enhanced proliferation and resistance to apoptosis of pulmonary artery smooth muscle cells (PASMCs) constituting vessel media. The sustainability of this phenotype is a result in part of activation of the transcription factor signal transducer and activator of transcription 3 (STAT3).^5^ Furthermore, other transcription factors and proteins have been identified as potential triggers of vascular dysfunctions and PA remodeling in PAH. Among these, peroxisome proliferator‐activated receptor gamma (PPARγ)^6–7^ and bone morphogenetic protein receptor 2 (BMPR2)^8–10^ have been shown to be decreased in PAH and to be part of the PAH remodeling process.

The receptor for advanced glycation end products (RAGE) is a member of the immunoglobulin protein family of cell‐surface molecules and has structural homology with other immunoglobulin‐like receptors.^11^ In the majority of healthy adult tissues, RAGE is expressed at a low basal level on different cell types such as macrophages, smooth muscle cells, endothelial cells, and cardiac myocytes,^12^ and its upregulation seems to be implicated in many pathological processes.^13^ First described in 1992, RAGE has attracted increasing attention because of its diverse ligand repertoire and its involvement in several pathological processes such as diabetes, cancer, renal and heart failure, and neurodegenerative diseases.^14^ We also demonstrated its role in systemic vascular remodeling by activating STAT3/provirus integration site for Moloney murine leukemia virus/nuclear factor of activated T‐cell cascade stimulating smooth‐muscle cell proliferation and resistance to apoptosis through a calcium‐dependent mechanism.^15^ Indeed, from a molecular point of view, RAGE seems to be implicated in many signaling pathway such as inflammation, proliferation, and migration,^16–19^ which are all implicated in PAH etiology. Furthermore, in their recent proteomic analysis, the Wilkins team demonstrated that RAGE is one of the most upregulated proteins in PAH lung tissues compared with control lung tissues,^20^ leading to our hypothesis that RAGE is implicated in PAH etiology.

S100A4 (also known as Mts1, metastasin, p9Ka, pEL98, CAPL, calvasculin, Fsp‐1, and placental calcium‐binding protein) belongs to the family of EF‐hand calcium‐binding proteins^21^ and is a strong RAGE agonist.^12,18,22^ S100 proteins regulate a variety of cellular activities^23^ and are known to be implicated in cancer and other proliferative diseases,^24^ as enhanced S100A4 expression is, in fact, a biomarker of poor prognosis in breast cancer.^23^ Increased expression of S100A4/Mts1 is observed PAH, contributing to smooth‐muscle cell proliferation and migration.^25^ Indeed, increased S100A4 levels are found in plexogenic lesions of the lungs of patients with severe PAH, which is not very surprising because of its angiogenic capacities.^26^ In PAH, studies have shown possible crosstalk between S100A4 and BMP proteins and receptors in PAH,^27^ thus implying a putative role of RAGE in this signaling pathway. New avenues demonstrate the potential role of STAT3 in decreased expression of BMPR2 in PAH.^28^ As mentioned, BMPR2 and PPARγ are implicated in PAH pathology, and some links seem to exist between these factors.^9^ Furthermore, in chondrocytes, RAGE can regulate PPARγ expression and activation.^29^ All these data led us to our hypothesis that RAGE activation by S100A4 triggers STAT3 activation, decreasing BMPR2 and PPARγ in pulmonary smooth‐muscle cells and leading to the proproliferative and apoptosis‐resistant phenotype found in PAH.

The present study aimed to elucidate the role of RAGE in PAH etiology because this receptor is widely overexpressed in PAH patients' lungs, is implicated in vascular remodeling, and could explain STAT3 activation as well as BMPR2 and PPARγ downregulation, known to be implicated in PAH pathobiology.

## Methods

All human tissues were obtained from the pneumology tissue bank of the Institut universitaire de cardiologie et de pneumologie de Québec (IUCPQ). All experiments were performed with Laval University and the IUCPQ Biosafety and Ethics Committee.

### Human Tissue Samples

All patients gave written informed consent before the study. Healthy lung tissues (controls) were obtained during lung resection for tumors. Only the healthy parts of the lungs were used in this study. PAH lungs were from lung explants from transplant. Brain, kidneys, and lungs were obtained from autopsy performed on PAH patients in which PAH was the cause of death. Age‐ and sex‐matched control and healthy (no diseases) tissues were obtained from autopsy of donor (at least 4 patients in each group for kidney and brain tissues; 8 patients in each group for lung tissues). In both cases, we selected patients in whom autopsy was performed within a few hours following death. Fresh human quadriceps biopsies were obtained from both PAH and age‐ and sex‐matched healthy donors (4 patients in each group) (Table 1).

**Table 1.** Patients Who Provided Tissues

	Patient Type	Sex	Age	Mean PA Pressure (mm Hg)	PVR (dyne×s)/cm^5^	Tissue Sample				Therapies
						Lung	Brain	Kidney	Quadricep	
1	Healthy	F	43	ND	ND	x				None
2	Healthy	F	38	ND	ND	x				None
3	Healthy	M	48	ND	ND	x				None
4	Healthy	M	45	ND	ND	x				None
5	Healthy	F	35	ND	ND	x				None
6	Healthy	M	51	ND	ND	x				None
7	Healthy	F	47	ND	ND	x				None
8	Healthy	F	50	ND	ND	x				None
9	Healthy	F	66	ND	ND		x	x		None
10	Healthy	F	52	ND	ND		x	x		None
11	Healthy	F	65	ND	ND		x	x		None
12	Healthy	F	59	ND	ND		x	x		None
13	Healthy	F	52	ND	ND		x	x		None
14	Healthy	M	72	ND	ND		x	x		None
15	Healthy	M	76	ND	ND				x	None
16	Healthy	F	45	ND	ND				x	None
17	Healthy	F	41	ND	ND				x	None
18	Healthy	F	30	ND	ND				x	None
19	iPAH	F	36	67	2274	x	x	x		Epoprostenol, furosemide, warfarin
20	iPAH	F	38	50	799	x				Epoprostenol, furosemide, sildenafil
21	iPAH	F	58	56	1709	x				Epoprostenol, furosemide, warfarin
22	PAH‐VOD	F	51	51	1199	x		x		Furosemide, warfarin
23	PAH‐VOD	F	63	59	926	x				Bosentan, furosemide
24	PAH‐VOD	M	72	39	948	x				Sitaxsentan, furosemide
25	PAH‐VOD	M	58	42	991	x				Epoprostenol, furosemide, warfarin
26	PAH‐VOD	F	48	73	1800	x				Epoprostenol, furosemide
27	iPAH	F	58	56	1709		x	x		Epoprostenol, furosemide, warfarin
28	PAH‐VOD	F	51	41	990		x	x		Furosemide
29	SSc‐PAH	F	55	48	980		x	x		Epoprostenol, furosemide, warfarin
30	Heritable PAH	F	55	66	1013.3				x	Bosantan, sildenafil, furosemide
31	iPAH	F	38	62	770.4				x	Furosemide
32	iPAH	F	44	33	373.3				x	Sildenafil
33	SSc‐PAH	F	55	27	188.9				x	Furosemide

PA indicates pulmonary artery; PVR, pulmonary vascular resistance; F, female; ND, not determined; M, male; iPAH, idiopathic pulmonary arterial hypertension; PAH‐VOD, pulmonary arterial hypertension associated with a veno‐occlusive disease; SSc‐PAH, pulmonary arterial hypertension associated with scleroderma.

### Cell Culture

For all PASMCs (control and PAH), we used cells in the fourth to sixth passages. PAH‐PASMCs were isolated from small pulmonary arteries from 3 patients with PAH, defined as a mean PA pressure of 25 mm Hg, as previously described.^30^ Control PASMCs from 4 patients were purchased (Cell Application Group, San Diego, CA). If >1 measure was taken on a patient's cell line, we confirmed that our results were reproducible and kept the mean per patient in our analysis to have n=3 to 5 cell lines per condition. PASMCs were transfected by CaPO_4_ precipitation with 20 nmol/L small interfering RNA (siRNA) oligonucleotides (siRAGE, siBMPR2, siSTAT3, or their negative control, siSCRM, all from Ambion). A final concentration of 100 ng/mL of S100A4 (Abcam) was used as a RAGE agonist, and PPARγ modulators (rosiglitazone and W9662, both from Cayman Chemical) were used at 1 μmol/L (all for a 48‐hour period).

### Luciferase Assay

Control PASMCs were transfected (100 000 cells per well in 24‐well plates) using CaCl_2_ with 20 nmol/L of siRNAs against RAGE or BMPR2. The next day, cells were transfected as previously described with 400 ng of a reporter plasmid containing 3 artificial binding sites for PPARγ and 80 ng of β‐galactosidase.^31^ Six hours later, transfection medium was removed, and fresh medium was added with the drugs (in concentration described above). Luciferase and β‐galactosidase activities were measured 24 hours later as described.^32^ Results are shown as luciferase activity relative to that of β‐galactosidase.

### Quantitative RT‐PCR and Immunoblots

These experiments were performed as previously described.^33–34^ Quantitative RT‐PCR (qRT‐PCR) 2^ΔΔCt^ was calculated with 18s as the housekeeping gene (Taqman Gene Expression Assay, Applied Biosystem, Foster, CA). For immunoblots, protein expression of RAGE (cell signaling), PPARγ (Santa Cruz), BMPR2 (Abcam), PY705‐STAT3, and STAT3 (the latter 2 both from cell signaling) were quantified and normalized amidoblack or ponceau as previously described.^34^ The PY705‐STAT3/STAT3 ratio evaluation was obtained from the same gel after 30 minutes' stripping at 50 degrees.

### Confocal Microscopy/Immunofluorescence

Sections of lungs and human PASMCs were used for immunofluorescence staining. For lung samples, only small and distal pulmonary arteries (<100 μm) were investigated. Rat lungs were fixed with 4% paraformaldehyde. Immunofluorescence was performed on 5‐μm lung slices. PASMC were fixed with 1% paraformaldehyde and permeabilized with 0.2% Triton X‐100. The same P‐STAT3, RAGE, PPARγ, and BMPR2 antibodies used with immunoblotting were used. Smooth‐muscle actin antibody was purchased from Sigma. Alexa Fluor 488 and ‐594 were used as secondary antibodies.

### Proliferation and Apoptosis Measurements

To study the effect of RAGE on PASMC proliferation and apoptosis in vitro, we established a model in which cultured human PAH‐PASMCs were exposed to 10% FBS (a condition known to promote proliferation^34–35^) or 0.1% FBS (a starvation condition that promotes apoptosis^34–35^). PASMC apoptosis and proliferation were measured using an Apoptag apoptosis detection kit (TUNEL; Millipore) and Ki67 antibody (Millipore), respectively. Percentage of nuclei‐positive PASMCs for TUNEL or Ki67 was determined and divided by the total amount of cells, calculated with 4′,6′‐diamidino‐2‐phenylindole (DAPI) (total nuclei).

### Animal Models

Male Sprague‐Dawley rats (250 to 350 g; strain 400; Charles River) were used for both animal models. For the monocrotaline‐induced PAH model (MCT), rats were injected subcutaneously with 60 mg/kg of crotaline (Sigma). Intratracheal nebulization of either siSCRM (1 nmol; Ambion) or siRAGE (1 nmol; Ambion) was given on day 15, when PAH signs had already appeared on echocardiography. For the Sugen‐hypoxia model, rats were injected with 20 mg/kg of SU5416 (Sigma) and put in hypoxia (10% O_2_) for 3 weeks. Chambers were opened twice a week for cleaning and replenishment of food and water. Oxygen concentrations were continuously monitored with blood gas analyzers. Rats were treated weekly with siRNA nebulization starting in week 7. All rats underwent hemodynamic and echocardiography studies on a weekly basis as previously described (after hypoxia for the Sugen model). Right heart catheterizations (closed chest) were performed using SciScence catheters. Direct PA pressures were measured in both monocrotaline and Sugen models before euthanizing. After euthanizing, lung and heart were retrieved, and pulmonary arteries were filled by direct injection of Microfil (Flow Tech, Carver, MA) into the main pulmonary artery *ex vivo* to form a vascular cast for investigations using micro‐CT (eXplore CT‐120 scanner; Gamma Medica, Inc, Northridge, CA). Lung perfusion and total volume were analyzed using Microview software and image processing with Osirix software. Perfusion calculation was made by percentage of artery signal volume of entire lung signal volume.

### Statistics

Data are presented as mean±SEM. Normality of our data was assessed by Shapiro–Wilk normality test. All our data were normally distributed (*P*>0.05). For comparison of 2 means, we used the unpaired Student *t* test, and for comparison of >2 means, we used 1‐way analysis of variance followed by the Tukey–Kramer posttest. *P*<0.001 (***),*P*<0.01 (**), and *P*<0.05 (*) were considered statistically significant. In cultured cell‐based experiments, “n” indicates the number of patients from which PASMCs were isolated and used for the experiment (n=5 controls and 3 PAH cell lines), and in the in vivo studies, “n” indicates the number of animals (rats) per group per experiment.

## Results

### RAGE Is Increased in Human and Experimental PAH

**Figure 1.**
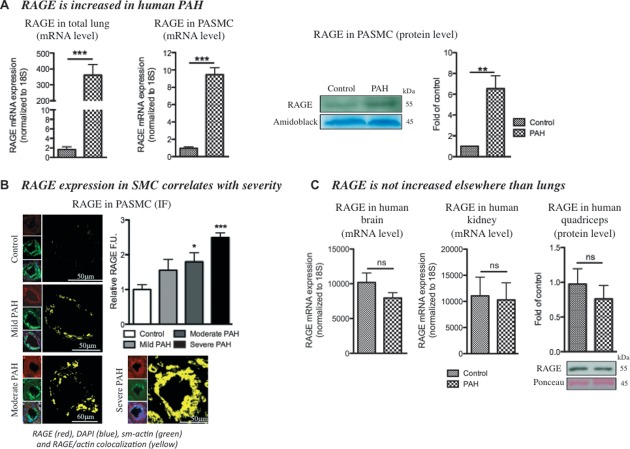
The receptor of advanced glycation end products (RAGE) is increased and activated in human pulmonary artery (PA) hypertension (PAH). A, RAGE expression measured by quantitative reverse‐transcription polymerase chain reaction (qRT‐PCR) normalized to 18S is significantly increased in both whole lung tissue (n=8 patients per group) and in PA smooth‐muscle cells (PASMCs) isolated from PAH patients compared with controls. This was confirmed by immunoblots showing a 6‐fold increase in PAH‐PASMCs compared with controls (n=3 PAH and n=5 control cell lines, *P*<0.01). B, RAGE protein expression in PASMCs of distal PAs (measured by immunofluorescence [IF] intensity) correlates with PAH severity (n=8 patients per group). Colocalization experiments between smooth‐muscle actin (red) and RAGE (green) gave greater yellow staining in distal PAs of patients with more severe PAH. C, RAGE mRNA and protein levels remain unchanged in brain, kidney, and peripheral muscle (quadriceps; n=4 patients per group).

To investigate the pattern of RAGE expression in normal and PAH lungs, we examined RAGE mRNA levels in total lung and RAGE protein levels in distal PAs from 8 individuals with nonfamilial PAH compared with 8 individuals without PAH (Figure 1A and Table 1). We found increased RAGE mRNA levels in human pulmonary hypertensive lung tissues compared with normotensive lung samples and increased RAGE protein levels in human pulmonary hypertensive distal PAs compared with normotensive distal PAs (>5‐fold increase, n=8 per group, *P*<0.01). Within the distal PAs, we found that the increase in RAGE protein level appeared earlier in the disease progression in PASMCs compared with endothelial cells and increased with disease progression (assessed by pulmonary vascular resistance), as shown by the greater amount of colocalization (yellow) in pulmonary hypertensive distal PAs between RAGE and smooth‐muscle actin rather than between RAGE and vascular endothelial (VE)‐Cadherin (Figures 1B and S1). As shown is Figure S1, RAGE was also increased in endothelial cells but at later stages of the pathology. To characterize whether RAGE upregulation is specific to the lung in PAH, we measured RAGE protein expression in human brain, kidney tissues, and quadriceps biopsies from healthy subjects and PAH patients (n=4 patients per group). As shown, no upregulation of RAGE was found in brain, kidney, or peripheral muscle (Figure 1C). As RAGE is mainly expressed in PAH‐PASMCs, we focused our research on PASMCs and provide evidence rather than in primary culture (<6 passages) of human PASMCs from healthy donors, and PAH patients' RAGE mRNA and protein expression was ≥5‐fold greater in PAH‐PASMCs than in control cells (n=3 to 5 cell lines, *P*<0.01; Figure 1A).

### RAGE Activation in Human PAH‐PASMCs Triggers the STAT3/BMPR2/PPARγ Axis

**Figure 2.**
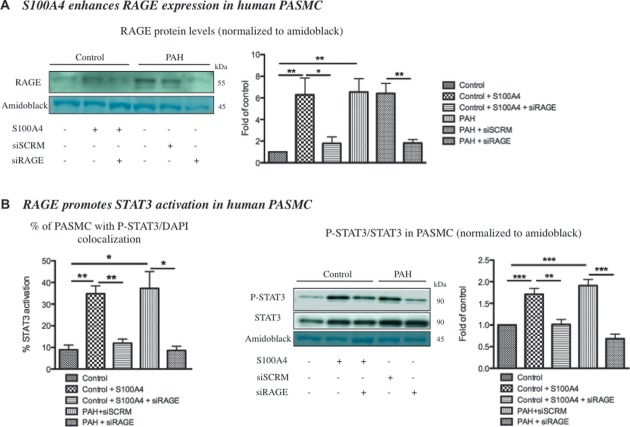
RAGE expression can be modulated in control and PAH‐PASMCs. A, Control PASMCs treated with S100A4 had a significant increase in RAGE expression measured by immunoblot (n=3 to 5 cell lines per group, *P*<0.05). It is also shown that scrambled siRNA (negative control, siSCRM) transfection had no additional effect on PAH‐PASMCs. B, This increase in RAGE expression and activation correlates with STAT3 activation measured in the exact same conditions by nuclear translocation assay (percentage of cells with PY705‐STAT3 nuclear localization) and immunoblot (PY705‐STAT3/STAT3 ratio normalized to amidoblack) (n≈50 cells/patient in 3 to 5 patients, *P*<0.05). RAGE indicates advanced glycation end products; PAH, pulmonary artery hypertension; PASMCs, PA smooth‐muscle cells; siRNA, small interfering RNA; siSCRM, scrambled siRNA; STAT3, signal transducer and activator of transcription 3; DAPI, 4′,6′‐diamidino‐2‐phenylindole; siRAGE, RAGE siRNA.

To determine whether S100A4 could enhance RAGE expression in control cells and trigger the downstream STAT3/BMPR2/PPARγ axis, we stimulated control human PASMCs with S100A4 (100 ng/mL, as previously described^36^). S100A4‐treated cells showed a sustained 5‐fold increase in RAGE expression measured by immunoblot (Figure 2A) and qRT‐PCR (Figure S3A) compared with control cells. Indeed, increase in RAGE expression in S100A4‐treated cells was comparable to levels found in PAH‐PASMCs (5‐fold increase in PAH‐PASMCs and stimulated control cells, n=3 to 5 cell lines, *P*<0.05; Figure 2A). This also confirms that scrambled siRNA transfection (negative control) does not modify RAGE expression compared with PAH cells alone.

STAT3 activation (nuclear translocation of Y705‐phosphorylated STAT3) was measured in PASMCs. Compared with controls, the percentage of cells presenting colocalization between P‐STAT3 and DAPI was significantly increased in PAH‐PASMCs (>2‐fold increase, n=3 to 5 cell lines, *P*<0.05; Figure 2B). Furthermore, S100A4‐treated cells also showed a 2‐fold increase in STAT3 activation (Figures 2B and S1B). These findings were confirmed by immunoblots measuring the PY705‐STAT3/STAT3 ratio normalized to amidoblack (a 50% increase, n=3 to 5 cell lines, *P*<0.01; Figure 2B). Compared with scrambled siRNA (siSCRM), RAGE siRNA (siRAGE) significantly reduced STAT3 activation in both stimulated control cells and PAH‐PASMCs, restoring STAT3 activation at control levels (2‐fold decrease, *P*<0.05).

**Figure 3.**
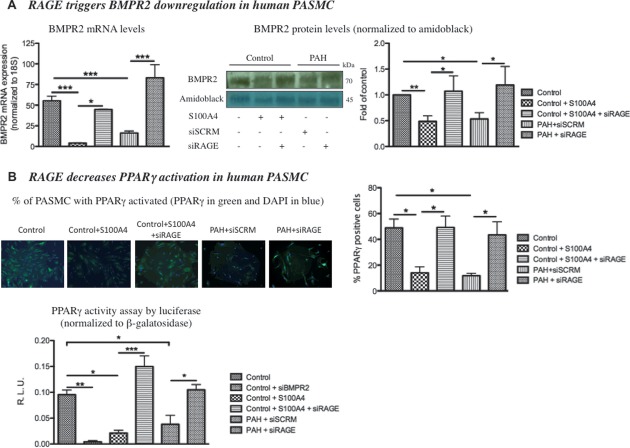
BMPR2 and PPARγ are known to be implicated in PAH, and we demonstrated that RAGE is upstream of these factors. A, BMPR2 mRNA and protein expression levels were measured in S100A‐treated cells and in PAH‐PASMCs with and without siRAGE. We demonstrated that BMPR2 is modulated with RAGE expression, as it was decreased in S100A4‐treated (100 ng/mL for 48 hours) and PAH cells and was restored on RAGE inhibition in both groups with both techniques (n=3 to 5 cell lines per group, *P*<0.05). B, Effect on PPARγ expression was measured by immunofluorescence (percentage of cells expressing PPARγ of total number of cells measured by DAPI; n≈50 cells/patient in 3 to 5 patients, *P*<0.05). These results were confirmed by the PPARγ luciferase assay (relative luminescence units; n=3 to 5 cell lines per group, *P*<0.05). Furthermore, we demonstrated that BMPR2 is upstream of PPARγ, as its inhibition (by siBMPR2) decreased PPARγ activity. In both stimulated control cells and in PAH‐PASMCs, RAGE inhibition increased PPARγ activity. BMPR2 indicates bone morphogenetic protein receptor 2; PPARγ, peroxisome proliferator‐activated receptor gamma; RAGE, advanced glycation end products; PAH, pulmonary artery hypertension; PASMCs, PA smooth‐muscle cells; siSCRM, scrambled siRNA; siRAGE, RAGE siRNA.

We also evaluated BMPR2 mRNA and protein levels in these same conditions. Control cells stimulated with S100A4 had decreased BMPR2 levels similar to what was found in PAH‐PASMCs (3‐fold decrease, n=3 to 5 cell lines; Figure 3A). The decrease in BMPR2 levels in S100A4‐stimulated and PAH cells was RAGE dependent, as BMPR2 levels were restored when cells were previously transfected with siRAGE measured at both the mRNA and protein levels (≥50% increase, n=3 to 5 cell lines, *P*<0.05; Figure 3).

Similar results were found with PPARγ expression and activation levels measured by immunofluorescence and luciferase assay. Control cells stimulated with S100A4 and PAH‐PASMCs had a 3‐fold decrease in PPARγ expression (percentage of cells with PPARγ activated of total cells measured by DAPI). This decrease was reversed on RAGE inhibition (2‐fold restoration; Figure 3B). Furthermore, to test whether RAGE has a direct impact on PPARγ endogenous transcriptional activity, a reporter gene construct containing 3 artificial PPARγ response elements upstream of the luciferase gene was transfected in PASMC control cells. PASMCs treated with S100A4 and cotransfected with this construct showed lower PPARγ transactivation than control cells (4× less, n=3 cell lines, *P*<0.05), and this effect was reversed on RAGE inhibition (siRAGE; 80% restoration). BMPR2 downregulation (siBMPR2) also decreased PPARγ activity, suggesting that PPARγ is a downstream target of this protein. PAH‐PASMC had lower PPARγ activity level (>2× less), which was increased with RAGE inhibition (≥50% increase, n=3 to 5 cell lines, *P*<0.05; Figure 3B). Note that RAGE siRNA efficiency was confirmed in both stimulated control PASMCs and in PAH‐PASMCs (Figure S3).

### RAGE Promotes PASMC Proliferation and Resistance to Apoptosis

**Figure 4.**
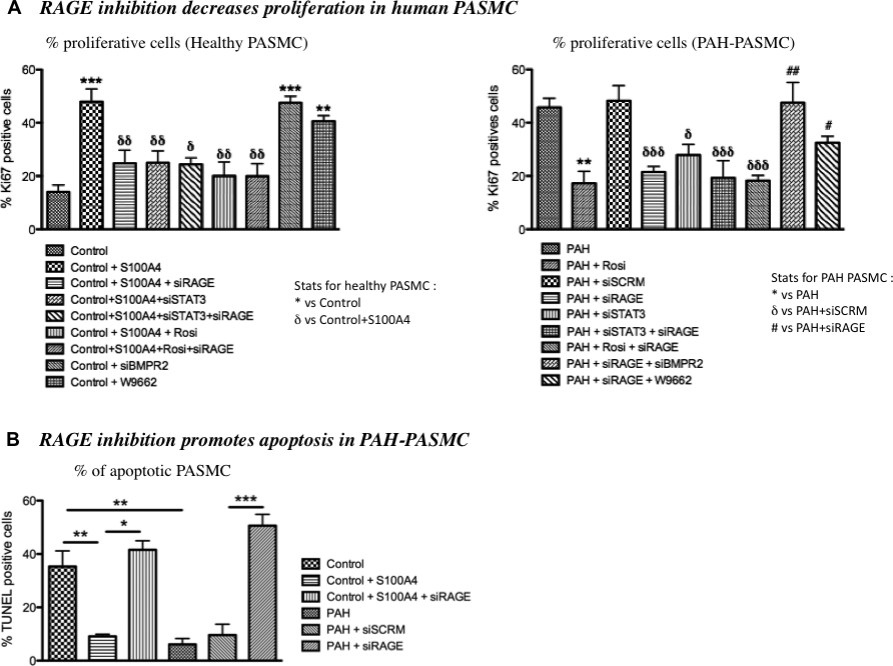
RAGE is implicated in the regulation of both pulmonary artery (PA) smooth‐muscle cell (PASMC) proliferation and apoptosis. A, Left, control PASMCs were stimulated with S100A4, W9662, or siBMPR2 with and without siRAGE, siSTAT3, and rosiglitazone (Rosi) treatment. S100A4‐treated cells had increased proliferation rates (percentage of Ki67‐positive cells; *P<0.001 vs control cells). This was reversed on RAGE (siRAGE) or STAT3 (siSTAT3) inhibition or PPARγ activation (rosiglitazone; δ*P*<0.05 vs S100A4). Decrease in BMPR2 (siBMPR2) or PPARγ (W9662) showed increased proliferation levels, like what was found with S100A4 (*P<0.01 vs control cells). Right, PAH‐PASMCs had increased proliferation, which was reversed on RAGE (siRAGE) or STAT3 (siSTAT3) inhibition (δ*P*<0.05 vs PAH+siSCRM) or PPARγ activation [rosiglitazone]; *P<0.01 vs PAH). The beneficial effect of RAGE inhibition was bypassed with BMPR2 (siBMPR2) or PPARγ (W9662) dowregulation (#*P*<0.05 vs PAH+siRAGE), demonstrating that they are downstream of RAGE (n≈50 cells/patient in 3 to 5 patients, *P*<0.05). B, S100A4‐treated and PAH‐PASMCs had decreased apoptosis (percentage of terminal deoxynucleotidyl transferase‐mediated dUTP nick‐end labeling [TUNEL]) and RAGE inhibition (siRAGE) significantly promoted apoptosis in both groups (n≈50 cells/patient in 3 to 5 patients, *P*<0.05). RAGE indicates advanced glycation end products; PAH, pulmonary artery hypertension; BMPR2, bone morphogenetic protein receptor 2; PPARγ, peroxisome proliferator‐activated receptor gamma; siSCRM, scrambled siRNA; siRAGE, RAGE siRNA; STAT3, signal transducer and activator of transcription 3.

To study the effect of RAGE on PASMC proliferation and apoptosis in vitro, we developed a model in which cultured human PAH‐PASMCs were exposed to 10% FBS to promote proliferation^34^ or 0.1% FBS to promote apoptosis,^34^ followed by ectopic delivery of RAGE siRNA (20 nmol/L for 48 hours). When compared with healthy PASMCs, PAH‐PASMCs expressing increased RAGE were characterized by a higher proliferation rate Figure 4A) and resistance to serum starvation‐induced apoptosis (Figure 4B)—increase of 20% in both proliferation and apoptosis resistance, n=3 to 5 cell lines, *P*<0.05. The implication of RAGE in regulating PASMC proliferation and apoptosis was confirmed in healthy PASMCs, in which RAGE activation by S100A4 increased proliferation and resistance to apoptosis to levels similar to those seen in PAH‐PASMCs (Figure 4). These results were reversed on RAGE inhibition by siRNA, demonstrating that this receptor is implicated in the proliferation and resistance to the apoptosis PAH‐like phenotype.

### RAGE Upregulation Promotes Activation of the Proproliferative and Antiapoptotic STAT3/BMPR2/PPARγ Pathway in PAH‐PASMCs

The increase in PASMC proliferation and resistance to apoptosis seen in PAH has been linked in part to the activation of STAT3, thus accounting for BMPR2 and PPARγ downregulation. This putative implication of RAGE was investigated in vitro by immunoblot, immunofluorescence, luciferase assays, and nuclear translocation assays. The activity of STAT3 (increased P‐STAT3/STAT3 ratio and P‐STAT3 nuclear translocation) was increased in PAH‐PASMCs, whereas both BMPR2 (immunoblot) and PPARγ were downregulated in PAH‐PASMCs (decreased expression—as seen from the lower percentage of green fluorescence and luciferase activity) (Figures 2 and 3). The increased activation of STAT3 was mediated through an interaction with RAGE, as RAGE siRNA decreased it in PAH‐PASMCs (Figure 2), and RAGE activation (S100A4) promoted it in healthy PASMCs (n=3 to 5 cell lines, *P*<0.05, Figure 2). These findings demonstrate that upregulation of RAGE accounts for activation of the STAT3/BMPR2/PPARγ pathways in PAH‐PASMCs. RAGE downstream targets have similar effects on PASMC proliferation rather than RAGE activation or inhibition. Indeed, in control cells, activation of RAGE (S100A4), and decrease of PPARγ (W9662) or BMPR2 (siRNA) induces a minimum 2‐fold increase in proliferation rates (Figure 4A). The S100A4 induced proliferation can be inhibited by RAGE or STAT3 inhibition (siRNAs) or PPARγ activation (rosiglitazone) demonstrating that RAGE, STAT3 and PPARγ are downstream effectors of S100A4. Furthermore, coinhibition of RAGE and STAT3, as well as inhibition of the RAGE simultaneously with PPARγ activation do not have synergic effects showing that these targets act in the same molecular pathway (Figure 4A). We also demonstrated that RAGE is upstream of BMPR2 and PPARγ by blocking RAGE, which decreases proliferation, and inhibiting BMPR2 (siRNA) or PPARγ (W9662), which confers a PAH proproliferative phenotype (increase ≥10% in proliferation rates, n=3 to 5 cell lines, *P*<0.05). These results confirm that BMPR2 and PPARγ are downstream targets of RAGE, as their inhibition bypasses RAGE blockade and thus increases proliferation compared with RAGE inhibition alone. Representative acquisitions can be found in Figure S2.

### RAGE Inhibition Reverses Both MCT‐ and Sugen‐Induced PAH

**Figure 5.**
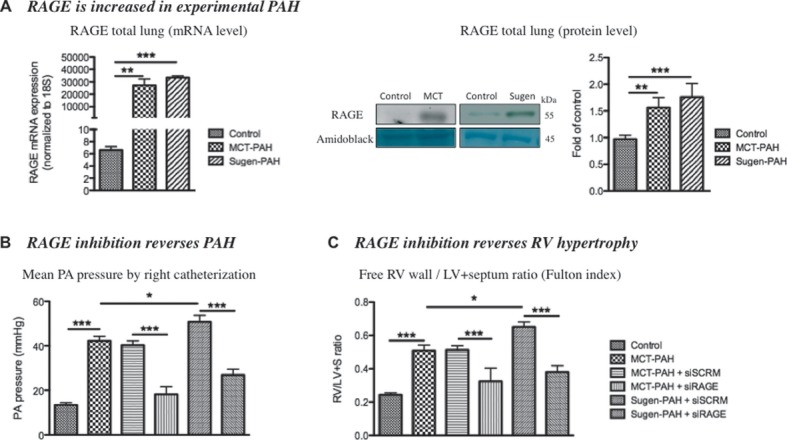
RAGE expression was increased in both MCT‐ and Sugen‐injected associated with hypoxia (Sugen) rat experimental PAH models, and RAGE inhibition (siRAGE nebulization) reversed PAH. A, Heavy RAGE mRNA and protein expression can be seen in lungs of rats with severe PAH (>28 days post‐MCT injection and >10 weeks post‐Sugen injection; n=8 rats per group). B, RAGE inhibition (1 nebulization of 1 nmol of siRAGE on day 15 for the MCT model and weekly nebulization of 1 nmol starting at week 7 in the Sugen model) decreased mean PA pressure. Sugen‐induced PAH was more severe than in the MCT‐induced model, as shown by mean PA pressure (n=8 rats per group, *P*<0.05). C, The Sugen model also had increased right ventricular (RV) hypertrophy compared with the MCT model. RAGE inhibition reversed RV hypertrophy (calculated by RV free wall on left ventricle [LV] and septum ratio) in both models (n=8 rats per group, *P*<0.05). RAGE indicates advanced glycation end products; MCT, monocrotaline; PAH, pulmonary artery hypertension; siSCRM, scrambled siRNA; siRAGE, RAGE siRNA.

To investigate the pattern of RAGE expression in normal and PAH lungs, we examined RAGE mRNA levels in total lung and RAGE protein levels in distal PAs from 5 rats with Sugen‐induced PAH compared with 5 control rats (Figures 5A and S3) and from 5 rats with monocrotaline‐induced pulmonary hypertension compared with 5 control littermates (Figures 5A and S4) by qRT‐PCR, immunoblots, and immunofluorescence (≥1.5‐fold increase, n=5 per group, *P*<0.01). As with in vitro, we observed that RAGE is upregulated in lungs and distal PAs. To test whether RAGE inhibition can reverse PAH symptom in these rat models, RAGE siRNA molecules were selectively delivered to the lungs of monocrotaline (MCT)–injected rats, an accepted model of PAH,^34^ as well as to the lung of the Sugen model (SU5416 injection and 3 weeks of hypoxia), a new model with more severe PAH and characteristics found in human PAH.^37^ Fifteen days after MCT injection or 7 weeks post–Sugen injection (when PAH is established), intratracheal nebulizations of RAGE siRNA (1 nmol/nebulization) were performed once for the MCT model and on a weekly basis until week 11 for the Sugen model (to maintain the inhibitory effect of the silencing RNA to week 12). We have extensively used this technique and previously shown its efficiency and specificity.^5^ This technique does not induce inflammatory cell infiltration or alveolitis (data not shown). Rats were monitored by echocardiography.^34^ A longitudinal study to assess our treatment's efficacy using noninvasive measurements was performed on a weekly basis (Doppler echocardiography). We observed that the local delivery of siRAGE in both MCT‐PAH and Sugen‐PAH rats reduced PA pressure assessed by PA acceleration time (PAAT), a Doppler parameter linked to PA pressure (PAAT being inversely correlated to PA pressure) and also decrease RV hypertrophy (n=8 rats per group, *P*<0.05; Figure S4). These data also demonstrate that at the time of treatment, PAH was already established in both models (PAAT significantly lower than in control rats and RV hypertrophy already present).

**Figure 6.**
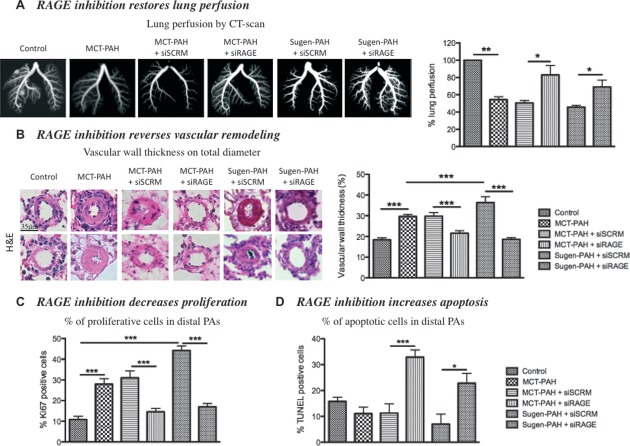
RAGE inhibition reverses vascular remodeling found in PAH. A, Lung perfusion was measured by CT scan, and decreased lung perfusion was found in both PAH models. RAGE inhibition increased lung perfusion in both models (n=8 rats per group, *P*<0.05). B, This correlated with distal PA remodeling, as PAH showed increased remodeling and RAGE inhibition diminished PA wall thickness (n≥8 rats per group, *P*<0.05). C, These effects were mediated by proliferation and apoptosis rates and, as with in vitro, RAGE inhibition decreased PASMC proliferation (percentage of Ki67‐positive cells) and increased apoptosis (percentage of TUNEL‐positive cells); n=5 arteries at random/rat with 8 rats per group, *P*<0.05. RAGE indicates advanced glycation end products; PAH, pulmonary artery hypertension; CT, computed tomography; PASMC, PA smooth‐muscle cell; siSCRM, scramble siRNA; siRAGE, RAGE siRNA; TUNEL, terminal deoxynucleotidyl transferase‐mediated dUTP nick‐end labeling.

**Figure 7.**
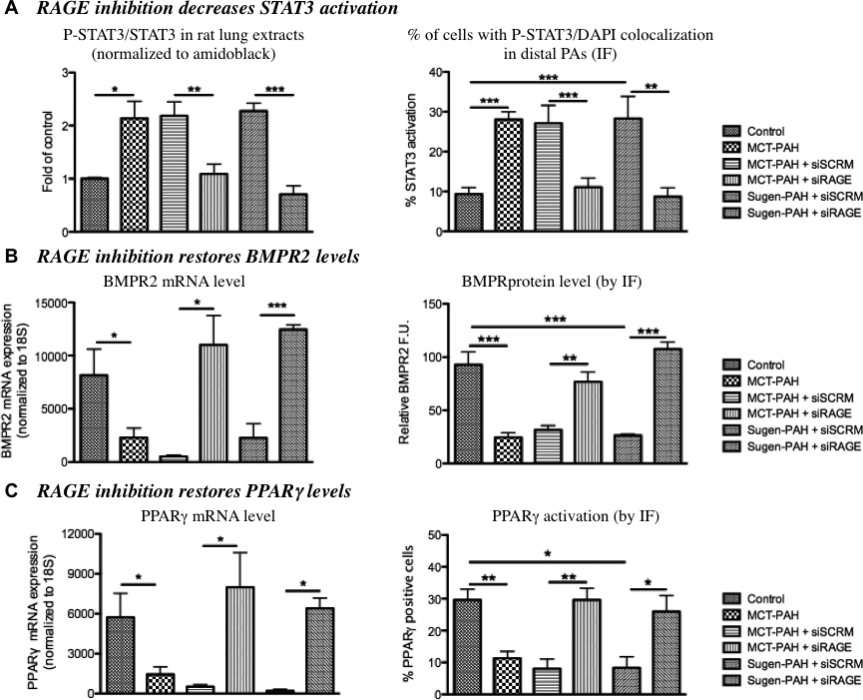
As with in vitro, proliferation was mediated by STAT3, BMPR2, and PPARγ, and RAGE inhibition restored whole signaling pathway. Thus, RAGE inhibition decreased STAT3 activation (PY705/STAT3 ratio by immunoblot and PY705‐STAT3 nuclear translocation by immunofluorescence), increased BMPR2 and PPARγ mRNA and protein expression (qRT‐PCR and immunofluorescence [IF]) in total lung extracts and in distal PA, respectively (for IF: n=5 arteries at random/rat with 8 rats per group; for qRT‐PCR and immunoblot: n=5 rats per group, *P*<0.05). STAT3 indicates signal transducer and activator of transcription 3; DAPI, 4′,6′‐diamidino‐2‐phenylindole; BMPR2, bone morphogenetic protein receptor 2; PPARγ, peroxisome proliferator‐activated receptor gamma; RAGE, advanced glycation end products; PAH, pulmonary artery hypertension; siSCRM, scrambled siRNA; siRAGE, RAGE siRNA; qRT‐PCR, quantitative reverse‐transcription polymerase chain reaction.

These findings were invasively confirmed by direct PA catheterization to precisely measure mean PA pressure and by the ratio of RV free wall weight over septum plus left ventricular (LV) free wall weight, as the index of RV hypertrophy (Fulton index) (Figure 5B and 5C). RAGE siRNA‐treated rats demonstrated a decrease >25 mm Hg in PA pressure and decreased RV hypertrophy, demonstrating the beneficial effect of RAGE inhibition in PAH not only on pressure (control rats have mPAP ≈15 mm Hg, PAH rats have mPAP >40 mm Hg, and with RAGE inhibition, mPAP decreases to 20 and 28 mm Hg, respectively, in MCT and Sugen models), but also on RV condition (35% decrease in RV hypertrophy, n=8 rats per group, *P*<0.001; Figure 5C). Furthermore, these data confirm that the Sugen‐induced PAH model is a more severe model than the MCT model, as mean PA pressure and RV hypertrophy were significantly higher in the Sugen model compared with the MCT model (n=8 per group, *P*<0.05). Moreover, lung perfusion was evaluated *ex vivo* using Microfil perfusion and CT scan analysis, demonstrating that RAGE inhibition restores blood flow through distal PAs, thus increasing lung perfusion (from 50% perfusion to 80% perfusion in the MCT model and from 45% to 75% in the Sugen model; Figure 6A). Vascular remodeling and vasoconstriction are accountable for blood flow restoration (measured by CT scan). Thus, we also performed an H&E coloration assay on harvested lungs to measure vascular remodeling (medial cross‐sectional area) in all groups. As expected, RAGE inhibition displayed a significant reduction in PA medial thickness in both models (decrease ≥10% in medial wall thickness, n=8 per group, *P*<0.001; Figure 6B), demonstrating that RAGE inhibition plays on the roots of the problem: the remodeling process and not only vasoconstriction. From a molecular point of view, as with in vitro, this was associated with increased PASMC proliferation in distal PAs (as assessed by Ki67; n=8 rats per group, *P*<0.05; Figures 6C and S5). RAGE inhibition decreased PASMC proliferation and increased apoptosis (TUNEL; Figures 6B and 6C and S5) in both models. RAGE inhibition in vivo, just like in vitro, reversed these phenotypes by decreasing STAT3 activation and restoring BMPR2 and PPARγ expression (by immunoblot, qRT‐PCR, and immunofluorescence; n=8 rats per group, *P*<0.05; Figures 7, S6, and S7). RAGE inhibition improved PAH in both animal models by providing beneficial effects on proliferation, apoptosis resistance, and the STAT3/BMPR2/PPARγ axis, thus making this protein a new potential therapeutic target for PAH.

## Discussion

**Figure 8.**
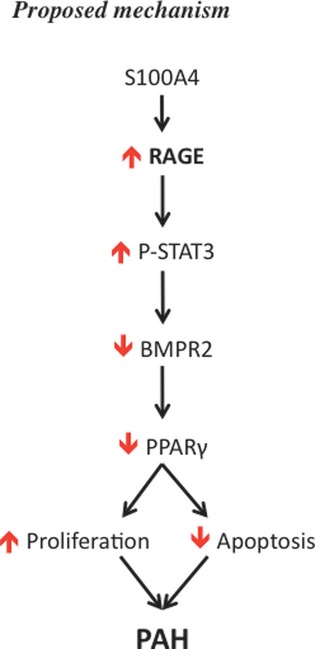
Proposed mechanism of the role of the receptor of advanced glycation end products (RAGE) in pulmonary arterial hypertension (PAH). Schematic representation of the RAGE/STAT3/BMPR2/PPARγ axis in PAH pathogenesis. Increased S100A4 triggers RAGE activation, resulting in STAT3 activation, decreased BMPR2 expression and PPARγ activation. Once this axis is activated, it increases smooth muscle cell proliferation and resistance to apoptosis, characteristic of PAH. RAGE indicates advanced glycation end products; STAT3, signal transducer and activator of transcription 3; PAH, pulmonary artery hypertension; PASMC, PA smooth‐muscle cells; BMPR2, bone morphogenetic protein receptor 2; PPARγ, peroxisome proliferator‐activated receptor gamma.

Here, we have demonstrated that RAGE is associated with PAH development, and we believe it to be a new avenue of investigation for PAH treatment. RAGE is overexpressed in PAH patients' lungs and is activated by S100A4, which is also increased in human PAH. We showed that RAGE accounts for STAT3 activation, as well as BMPR2 and PPARγ downregulation (Figure 8).

We provided direct in vitro and in vivo evidences showing that the mechanism by which RAGE inhibition reverses PAH involves inhibition of PASMC proliferation within remodeled PA and restoration of BMPR2 and PPARγ expression and activation in the vessel wall. Because our goal was to identify a new way of reversing established PAH, we focused on PASMCs and not on endothelial cells, which seem to be more affected at the onset of PAH. Nonetheless, the beneficial effect of RAGE in the Sugen‐induced PAH model shows that RAGE may also play a role in endothelium‐related vascular lesions, such as plexiform lesions, which can be seen in PAH patients and in this experimental model. Further investigation will be necessary to evaluate the role of RAGE in the endothelial dysfunctions found in PAH.

RAGE is an interesting therapeutic target because of its role in PAH development. Indeed, we have evidence that STAT3 is activated before PAH development,^5,38^ and because RAGE accounts for STAT3 activation, it is likely critical in PAH development.

Different signaling mechanisms could explain the crosstalk between BMPR2 and PPARγ.

Indeed, in rabbit chondrocytes, RAGE downregulates PPARγ expression through the mitogen‐activated protein kinases (MAPKs) p38 and JNK.^29^ This mechanism could also take place in PAH because MAPK is implicated in cell migration in pulmonary hypertension.^39^

Also, newly investigated microRNA could have a potential role in this signaling pathway, as Dr Hart's team demonstrated the implications of miR17‐92 and miR21 in PPARγ regulation,^40^ and we also demonstrated that miR‐204 could indirectly, through Src kinase activity, modulate BMPR2 expression.^38^ Marx et al demonstrated that miR21 was increased in human PAH‐PASMCs and that PPARγ ligands attenuate these microRNA alterations. This could also explain the restoration of PPARγ mRNA levels with RAGE inhibition in our model.

Moreover, thiazolidinediones such as rosiglitazone have been shown to reduce endothelial RAGE expression in patients with diabetes,^41^ suggesting a possible feedback loop in our model, as we demonstrated that RAGE is an upstream mediator of PPARγ. More investigation will be needed to confirm this hypothesis in PAH, as some mechanisms are very different between pulmonary and systemic vasculature.

Finally, that pulmonary circulation is selectively diseased in human PAH is a major therapeutic challenge. The majority of drugs targeting the vasculature, if given systemically, will also affect healthy normal circulation, thereby limiting efficacy. In PAH, downstream targets of RAGE cannot be therapeutically targeted, as STAT3, even if implicated in vascular remodeling, is constitutively expressed in several tissues and implicated in the immune response.^42^ Also, recent studies showed that restoring BMPR2 levels does not reverse PAH,^43^ and, furthermore, clinical studies demonstrated that PPARγ agonist have adverse cardiovascular effects.^44^ Thus, a RAGE antagonist could be a potential treatment for PAH, because it showed no toxic effect in a phase II clinical trial for Alzheimer's disease^45^ and because RAGE inhibitors are clinically available. In addition, RAGE is one of the most overexpressed proteins in PAH patients' lungs,^20^ and this receptor is expressed at very low levels in normal adult cells,^13^ suggesting specific effects of a RAGE inhibitor. This project could be an interesting starting point to do preclinical trial and also to evaluate if RAGE could also be a PAH biomarker.

## Conclusions

Our results demonstrated that inhibiting RAGE reverses PAH even in an experimental model with severe PAH. Also, as mentioned, RAGE inhibitors already exist and are in clinical trials for Alzheimer's disease,^45^ in which they seem to be safe and well tolerated. Therefore, we suggest RAGE as a novel therapeutic target for PAH treatment because of its overexpression compared with control lungs and its implication in the pulmonary vascular remodeling process.
